# Evaluation of the immature platelet fraction contribute to the differential diagnosis of hereditary, immune and other acquired thrombocytopenias

**DOI:** 10.1038/s41598-017-03668-y

**Published:** 2017-06-13

**Authors:** F. L. B. Ferreira, M. P. Colella, S. S. Medina, C. Costa-Lima, M. M. L. Fiusa, L. N. G. Costa, F. A. Orsi, J. M. Annichino-Bizzacchi, K. Y. Fertrin, M. F. P. Gilberti, M. C. Ozelo, E. V. De Paula

**Affiliations:** Faculty of Medical Sciences/Hematology and Hemotherapy Center, University of Campinas, Campinas, SP Brazil

## Abstract

The differential diagnosis of immune (ITP) and hereditary macrothrombocytopenia (HM) is key to patient management. The immature platelet fraction (IPF) represents the subset of circulating platelets with higher RNA content, and has been shown to distinguish hypo- from hyperproliferative thrombocytopenias. Here we evaluated the diagnostic accuracy of IPF in the differential diagnosis between HM and other thrombocytopenias in a population of patients with post-chemotherapy thrombocytopenia (n = 56), bone marrow failure (n = 22), ITP (n = 105) and HM (n = 27). TPO levels were also measured in HM and ITP matched for platelet counts. Platelet counts were similar in all patient groups. Higher IPF values were observed in both ITP (12.3%; 2.4–65.6%) and HM (29.8%; 4.6–65.9%) compared to hypoproliferative thrombocytopenias. IPF values were also higher in HM compared to ITP, yielding a diagnostic accuracy of 0.80 (95%CI 0.70–0.90; P < 0.0001) to distinguish these two conditions. Intra- and inter-assays reproducibility of IPF in HM patients revealed that this is a stable parameter. In conclusion, IPF is increased in HM compared to both ITP and other thrombocytopenias and contributes to the differentiation between ITP and HM. Further studies are warranted to understand the biological rationale of these findings and to its incorporation in diagnostic algorithms of HM.

## Introduction

The differential diagnosis of thrombocytopenias includes a variety of conditions such as hematologic malignancies, bone marrow failure (BMF), hypersplenism, immune thrombocytopenia (ITP), microangiopathic hemolytic anemias and hereditary macrothrombocytopenia (HM). Among these, the differential diagnosis between ITP and HM can be challenging due to the absence of specific tests, particularly in patients with mild bleeding symptoms^1–3^. Recently, the feasibility of using parameters of the complete blood count (CBC) to support this differential diagnosis was illustrated by a series of studies which demonstrated and validated that the mean platelet volume (MPV) can help the segregation of patients with ITP and HM^4, 5^.

In recent years, new parameters were incorporated to the CBC, including the immature platelet fraction (IPF), which represents a population of newly formed platelets containing a greater amount of residual RNA^6^. Initially, the IPF was measured by flow cytometry, and described as reticulated platelets^7^. Recently, studies have reported the clinical utility of measuring immature platelets in clinical settings using automated hematology analyzers^8–10^. The correlation between flow cytometry and hematology analyzers in quantifying this population has been previously demonstrated^11^.

Although the utility of the IPF in the differential diagnosis between hypo- and hyperproliferative thrombocytopenias has been already reported^8, 12^, less information is available about its use in the differential diagnosis between ITP and HM. In 2000, Fabris *et al*. evaluated reticulated platelets by flow cytometry in a population of 29 patients with HM, observing reduced IPF values when compared to ITP^13^. More recently, Miyazaki *et al*.^14^, in a study with 15 patients with HM in which the IPF was measured in an automated hematology analyzer observed significantly higher IPF values in HM compared to ITP. To further elucidate the value of IPF determination in the diagnosis of thrombocytopenias, with focus on the differential diagnosis between ITP and HM, we investigated the diagnostic accuracy and the precision of IPF measurements in a population of patients with different causes of thrombocytopenia, including a well-characterized cohort of patients with HM. In addition, we also evaluated whether thrombopoietin (TPO) levels could further facilitate this diagnosis.

## Methods

### Study design and patient population

This was a cross-sectional diagnostic accuracy study, designed according to STARD (Standards for the Reporting of Diagnostic accuracy studies) guidelines^15^. The study population consisted of patients with thrombocytopenia confirmed in two independent samples and microscopic analysis, in regular clinical follow-up in the Hemostasis outpatient clinic of University of Campinas, or admitted to the hematology ward of the same institution. The inclusion criteria was a confirmed diagnosis of any of the following: (i) ITP (based on previously established guidelines)^16^; (ii) bone marrow failure (including aplastic anemia and myelodisplatic syndromes with a platelet count below 150 × 10^9^/L)^17, 18^; (ii) post-chemotherapy (Ctx) thrombocytopenia (in admitted patients with hematological malignancies) with a platelet count below 150 × 10^9^/L; and (iv) HM, with platelet counts below 150 × 10^9^/L. Diagnosis of HM was established by clinical and laboratory criteria as determined by international guidelines^19–21^. These included the exclusion of all other causes of thrombocytopenia, objective confirmation of thrombocytopenia in first-degree relatives, platelet aggregation studies, molecular analyses and specific tests such as platelet glycoprotein studies and electron microscopy in selected cases. Exclusion criteria included: a platelet count above 150 × 10^9^/L in ITP patients at the day of enrollment; (ii) the presence of conditions known to influence IPF values such as sepsis and other inflammatory diseases^22^, or (iii) the use of antiplatelet agents. The study was approved by the Institutional Review Board of University of Campinas (certificate number 411.620/2013) and performed in accordance with the Declaration of Helsinki. All patients provided written informed consent prior to enrollment.

Recruitment occurred between July 2013 and February 2015. ITP, HM and BMF patients were included consecutively, aiming for an enrollment of 100 patients with ITP, 20 with BMF and all patients with HM. Post-Ctx patients were included using a convenience sampling strategy, which consisted in weekly visits to the hematology ward, with a target of 50 patients.

### Sample collection and processing

Samples were collected by venipuncture by the same nursing team responsible for the collection of routine clinical samples, and using the same standard operating procedures in both clinical sites. Blood was drawn in Vacutainer® EDTA K2 tubes (Becton Dickinson-BD, Franklin Lakes, Nova Jersey, EUA). IPF measurements were performed within four hours from sample collection, which is the threshold suggested in the equipment operational procedures. After IPF analysis, tubes were centrifuged (2,500 g for 10 min at room temperature), and plasma aliquots were frozen at −80 °C until analysis.

### IPF measurement

Whole blood samples were analyzed in a Sysmex XE 5000 hematology analyzer (Sysmex, Kobe, Japan). Briefly, the IPF fraction was identified in the reticulocyte channel, employing a fluorescent dyes containing polymethine and oxazine. These dyes penetrate the cell membrane, staining residual RNA of red blood cells (reticulocytes) and platelets. In addition, these two populations are separated by cell size. A computer algorithm then discriminates mature platelets and the IPF, which was expressed as a percentage of total platelets (IPF%). In addition, we also calculated the absolute count of the IPF (A-IPF), which has been previously reported as superior to the IPF% in some clinical settings^9, 10^. Finally, we also evaluated the intra- and inter-assay reproducibility of IPF measurements in a subgroup of patients with HM. Platelet counts and mean platelet volume (MPV) were obtained from the same samples, by the same hematological analyzer, which measures the MPV by impedance. In the laboratory where IPF measurements were performed, commercial internal quality control samples (Sysmex e-Check, XE) are run before the beginning of each 8 h-routine, as well as after any corrective or preventive maintenance of the hematological analyzer. In addition, the laboratory participates in a regular national external quality control program and is within the scope of the University laboratory quality management system.

### TPO measurement

Circulating TPO levels were measured in plasma samples using a commercial immunoassay (Quantikine, “Human thrombopoietin Immunoassay”, R&D Systems, Minneapolis, USA), based on a sandwich ELISA technique, according to the manufacturer’s protocol, in all HM patients and in seventy-four ITP patients. Of note, none of these patients were using TPO receptor agonists. A group of eight patients with a history of ITP diagnosis that were not excluded from the study due to platelet counts above 150 × 10^9^/L were used as a control group for the measurement of TPO levels. All of these patients were off-ITP treatment for at least one year and had platelet counts above 250 × 10^9^/L at the time of testing.

### Statistical Analysis

Results are expressed as median and range, or means ± SD, unless mentioned otherwise. Differences between quantitative variables were evaluated by Mann-Whitney or Kruskal-Wallis with Dunn’s post-test for comparison with 2 or more than 2 variables respectively. Associations involving categorical clinical variables were analyzed using the Fisher exact test. Correlation analyses were performed using the Pearson or Spearman correlation (rho) test, according to data distribution. The diagnostic accuracy of IPF for the segregation of patients by each diagnosis was estimated by the ROC (receiver operating characteristic) procedure, which allows the simultaneous analysis of sensitivity and specificity of each test in relation to selected clinical outcomes. Results are reported with confidence intervals and level of significance. A P value < 0.05 was considered statistically significant.

## Results

Two-hundred forty-eight patients were enrolled in this study, of which 38 were excluded due to platelet counts above 150 × 10^9^/L (all of them from the ITP group). In total, 210 patients were analyzed, divided in the following subgroups: Post-Ctx (n = 56), BMF (n = 22), ITP (n = 105) and HM (n = 27). Demographic and clinical characteristics are shown in Table 1. For patients with HM, these data are shown individually (Table 2). In agreement with previous reports, the MPV was not reported by the analyzer used in this study in most patients with HM (Table 1) owing to abnormalities in platelet distribution curves^4, 5^. In all other samples, IPF was normally reported. Of note, no differences were found in platelet counts between ITP and HM patients.

**Table 1:** Patient characteristics.

	Post-Ctx (n = 56)	BMF (n = 22)	ITP (n = 105)	HM (n = 27)
Age*	51 (24–75)	64 (22–90)	55 (16–87)	29 (04–55)
Sex (male:female)	31:25	14:8	33:72	8:19
Platelet count (X 10^9^/L)*	31 (5–146)	27 (4–146)	52 (3–150)	52 (6–128)
MPV (fl)^†^	10.5 (±1.06)	9.2 (±2.15)	11.3 (±2.59)	—^††^

Post-Ctx: post-chemotherapy BMF: bone marrow failure; ITP: immune thrombocytopenia; HM: hereditary macrothrombocytopenia; MPV: mean platelet volume; *median (min-max); ^†^mean (±standard deviation); ^††^MPV was not measured in 20/27 of the HM patients.

**Table 2:** Characteristics of patients with hereditary macrothrombocytopenia.

UPN	Age/sex	Diagnosis	Platelet count (X 10^9^/L)	IPF (%)
1	33/M	MYH9	21	62.7
2	10/F	MYH9	6	59.8
3	24/F	MYH9	49	56.4
4	15/M	MYH9	52	25.5
5	17/F	MYH9	69	24.7
6	17/F	MYH9	20	43.2
7	12/F	MYH9	59	22.4
8	55/M	MYH9	12	62.7
9	42/F	MYH9	74	38.8
10	37/F	BSS	19	38.3
11	28/F	BSS	60	28.3
12	33/M	BSS	96	37.2
13	4/F	BSS	22	57.2
14	27/M	BSS	41	31.1
15	16/F	BSS	14	61.4
16	37/F	BSS	9	65.9
17	48/F	HMT	65	29.8
18	23/M	HMT	128	10.9
19	13/F	HMT	17	45.9
20	16/F	HMT	59	14.9
21	33/M	HMT	56	14.7
22	32/F	HMT	82	27.8
23	53/F	HMT	76	24.6
24	54/F	HMT	93	5.0
25	36/F	HMT	41	4.6
26	33/F	HMT	89	17.5
27	40/M	HMT	7	51.7

UPN: unique patient number; MYH9: *MYH9*-related platelet disorders; BSS: Bernard Soulier syndrome; HMT: non-specified hereditary macrothrombocytopenia; MPV: mean platelet volume; IPF: immature platelet fraction. F: female; M: male.

**Figure 1:**
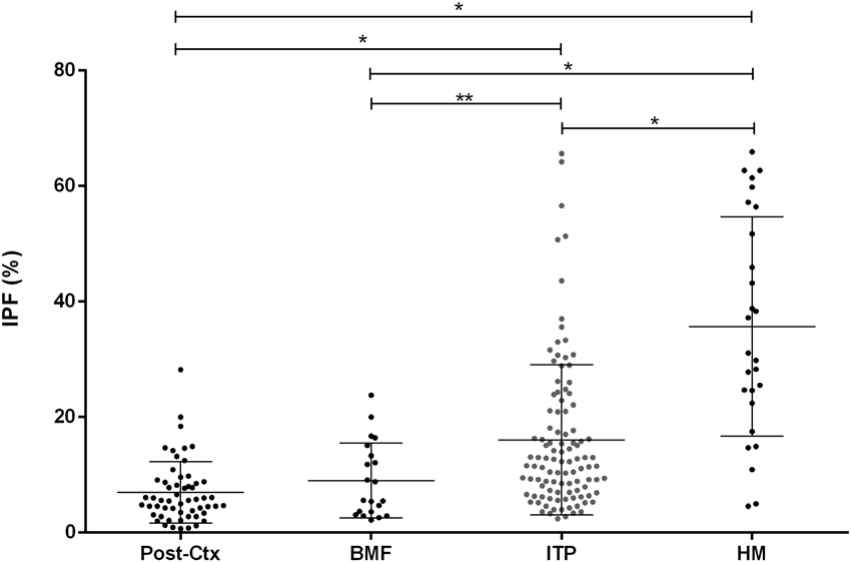
Immature platelet fraction (%) of patients with different causes of thrombocytopenia; Post-Ctx: post chemotherapy; BMF: bone marrow failure; ITP: immune thrombocytopenia; HM: hereditary macrothrombocytopenia. Kruskal-Wallis test with Dunn’s post test (**P < 0.001, *P < 0.05).

Similar IPF values were observed between patients with Post-Ctx thrombocytopenia and BMF. All other comparisons yielded significant differences in IPF between different patient groups (Fig. 1), with both ITP and HM patients with higher IPF levels than Post-Ctx and BMF. In addition, HM patients presented a significantly higher median IPF level (29.8%; 4.6–65.9%) than ITP patients (12.3%; 2.4–65.6%; P < 0.0001). Similar results were obtained when the IPF was analyzed in absolute values (A-IPF) (data not shown).

**Figure 2:**
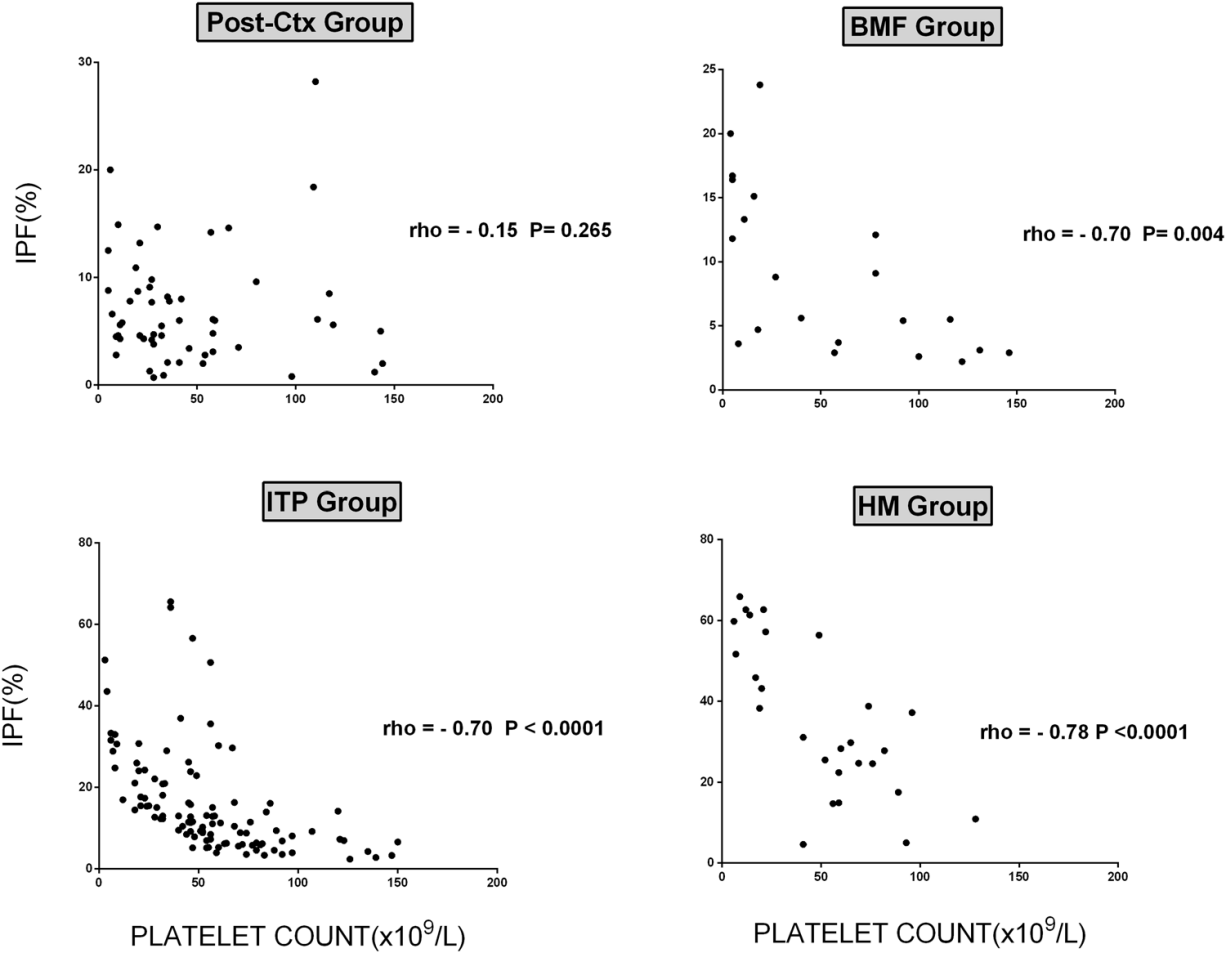
Scatter plots showing correlation between immature platelet fraction (IPF%) and platelet counts (X 10^9^/L) of patients with different causes of thrombocytopenia. (**a**) Post-chemotherapy, (**b**) bone marrow failure, (**c**) immune thrombocytopenia, (**d**) hereditary macrothrombocytopenia. Spearman correlation coefficient is shown.

An inverse correlation between IPF and platelet counts was observed in BMF, HM, and ITP, but not in Pos-Ctx patients (Fig. 2). IPF values correlated with MPV only in Post-Ctx (rho = 0.57; P < 0.0001).

The diagnostic accuracy of IPF% and A-IPF measurements for the differential diagnosis of thrombocytopenia was estimated using ROC analysis. The AUC for the differential diagnosis between ITP and HM was 0.80 (95%CI 0.70–0.90; P < 0.0001). Using an arbitrary IPF% cut-off value of 17.4%, which presented the best combination of sensitivity and specificity, ITP could be distinguished from HM with a sensitivity of 70% and a specificity of 90%. The positive and negative predictive values were 81.48% and 71.43 respectively. A similar result was obtained using the A-IPF, which yielded and AUC of 0.77 (95%CI 0.67–0.87; P < 0.0001). Therefore, all additional analyses were performed using the IPF%.

**Figure 3:**
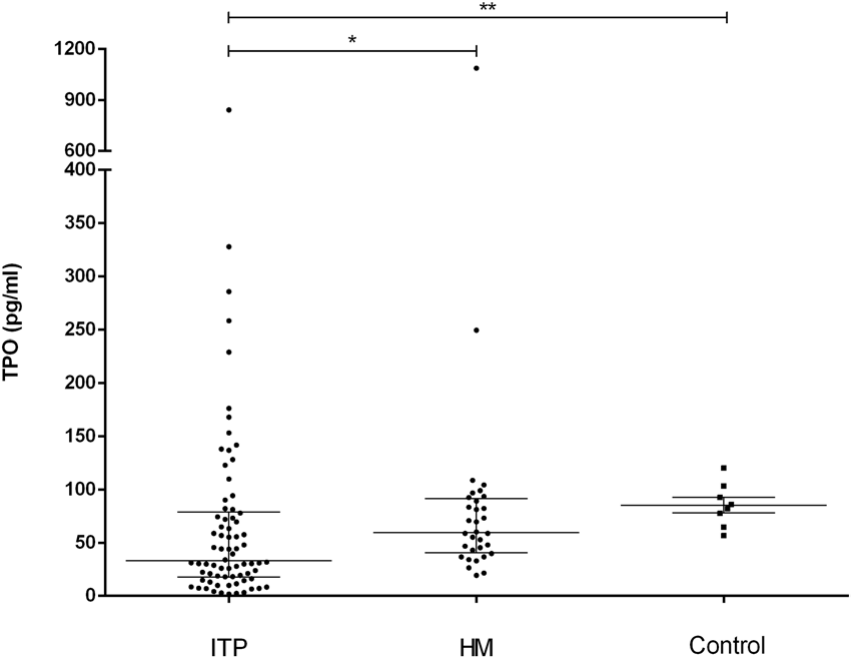
Thrombopoietin (TPO) levels were measured by ELISA in patients with immune thrombocytopenia (ITP; n = 74) and hereditary macrothrombocytopenia (HM; n = 27). Eight ITP patients in complete remission for at least one year and off any treatment were evaluated as a control population. Horizontal bars represent: medians and interquartile ranges. Kruskal-Wallis test with Dunn’s post test; *P = 0.04 and **P = 0.03.

As previously described, ITP patients had significantly lower TPO levels compared to control subjects^23^ despite the former group having much lower platelet counts than the latter. Even when compared to HM patients with similar levels of thrombocytopenia, ITP patients presented significantly lower TPO levels (Fig. 3). No significant correlation could be observed with platelet counts or IPF% in HM. Interestingly, despite lower TPO levels in ITP significant correlations were observed between TPO levels and platelet count (rho = −0.50; P < 0.001) and with IPF% (rho = 0.44; P < 0.001). The estimated diagnostic accuracy of TPO measurement for the differential diagnosis between ITP and HM was 0.66 (95%Cl 0.56–0.77; P = 0.007).

The intra- and inter-assay reproducibility of IPF% measurements were evaluated in six HM patients, all of them with IPF% values in the upper range of the IPF% distribution (Table 3). The coefficient of variation (CV%) of three sequential IPF% measurements (intra-assay reproducibility) was 5.4 ± 3.1, similar to the CV% of platelet counts from the same samples. In addition, we repeated the IPF% measurements of these patients after 12 months from the first measurement (Table 3). While platelet counts fluctuations were observed between these two measurements in some of these patients (CV% = 30.2 ± 29.0), IPF% remained fairly constant (CV% = 7.6 ± 3.9), suggesting that this is a stable parameter in HM, irrespective of fluctuations in platelet counts.

**Table 3:** Reproducibility of IPF measurements in patients with HM.

#	Intra-assay (same sample)				Inter-assay (12-month apart samples)			
	IPF*	CV%	Platelet*	CV%	IPF**	CV%	Platelet**	CV%
**1**	65.6	7.8	7.3	15.7	64.2	3.2	14.2	68.2
**2**	42.1	1.7	105.0	1.0	39.7	8.8	100.5	6.3
**3**	65.8	7.0	16.3	15.4	62.7	7.0	11.2	65.4
**4**	29.4	6.2	69.7	5.4	27.0	12.5	72.8	6.1
**5**	65.8	1.2	41.0	2.4	61.1	10.9	45.0	12.6
**6**	65.6	8.4	8.7	6.7	64.2	3.2	10.3	22.8
**Total**	55.7 ± 16.0	5.4 ± 3.1	41.3 ± 39.3	7.8 ± 6.4	53.2 ± 15.9	7.6 ± 3.9	42.3 ± 37.7	30.2 ± 29.0

Intra-assay reproducibility was measured by 3 sequential measurements of IPF% in the same sample, with *indicating the mean of these 3 results. The inter-assay reproducibility was measured by repeating the IPF% in the same patient after 12 months, with **indicating the mean of these 2 results. Total represents the mean and SD of six patients.

Despite the low number of patients with HM, we performed a subgroup analysis to investigate whether IPF values differed between patients with different types of HM (Table 2). While no difference was observed between patients with molecularly confirmed *MYH9*-related platelet disorders (IPF = 43.2%, range 22.4–62.7%) or Bernard-Soulier syndrome (IPF = 38.3%, range 28.3–65.9%), lower IPF values were observed in the remaining patients classified as non-specified hereditary macrothrombocytopenia (IPF = 17.5%, range 4.6–51.7%; P < 0.05).

## Discussion

The differential diagnosis between ITP and HM represents a significant clinical problem since tests used to distinguish these two conditions are not available in most clinical laboratories, and even when available, cannot discern among all clinical etiologies. The development of hematological analyzers in the last decades has allowed the incorporation of new parameters in the diagnosis of hematological disorders; and the feasibility of this strategy for the diagnosis of HM has been shown by a series of studies evaluating platelet size in these patients. These studies demonstrated that MPV is significantly higher in patients with HM compared to ITP^4^, a finding that was later validated in a multicenter study using different instruments to measure this parameter^5^. Using a population of 210 patients with different causes of thrombocytopenia, the main result of our study was that another platelet parameter derived from automated an hematology analyzer reliably differentiates HM from ITP, with an estimated diagnostic accuracy similar to that reported in the literature for MPV^4, 5^.

In our population, IPF% values were significantly higher (almost three times) in the HM group compared to hypoproliferative thrombocytopenias such as BMF and Post-Ctx. More importantly, IPF% values were higher in HM than ITP patients with similar platelet counts. An inverse correlation between platelet counts and IPF% was observed not only in ITP patients but also in patients with BMF and HM. The absence of such correlation in patients with Post-Ctx thrombocytopenia is most likely due to the blunted thrombopoietic response secondary to chemotherapy. The ROC analysis estimated significant diagnostic accuracies of IPF% and A-IPF in the differential diagnosis of ITP and HM even in a group of patients with similar levels of thrombocytopenia (median 52 × 10^9^/L for both groups). It should be noted that in contrast to other clinical settings, no relevant difference was observed in when IPF was reported as a percentage or in absolute values.

The increase of IPF in patients with hyperproliferative thrombocytopenias such as ITP has been recognized since the original demonstration that the RNA content of circulating platelets could be used as an indicator of thrombopoietic activity^24^. Formerly measured by flow cytometry, the evaluation of these recently produced platelets has been reported in other patient populations with different thrombocytopenic etiologies^12^. This was first assessed in 2000 in HM, in a study with 29 patients with chronic hereditary thrombocytopenia and 23 patients with ITP. Using a flow cytometry-based method, markedly lower reticulated platelet counts were observed in patients with HM compared to ITP^13^. More recently, the IPF% was evaluated in a group of 15 patients with congenital macrothrombocytopenia using an automated hematological analyzer as in our study. In contrast to Fabris *et al*., markedly higher IPF% levels were observed in HM patients compared to ITP patients^14^. Herein, we confirm and extend these results in an independent and larger population of 27 well-characterized patients with HM and 105 patients with ITP. The validation of IPF% measurement as a diagnostic tool in HM is important due to differences in the management of these patients compared to ITP and the current lack of accessible tests for this purpose. Moreover, our study found good intra- and inter-assay reproducibility of IPF% measurements in patients with HM. This is particularly important due to the observation of Myazaki *et al*., that small platelet aggregates could increase IPF% values^14^, and to the fact that mechanisms underlying the increase in IPF% values in these patients have not been elucidated. In this context, the low CV of IPF% measurements both in the same sample and in different samples from the same patient support that the IPF% in HM is a stable and robust indicator, paving the way for studies aimed to evaluate its incorporation in the clinical evaluation of these patients.

We also evaluated whether TPO measurements could contribute to the differential diagnosis between HM and ITP, or provide insights about the mechanisms underlying the variation of IPF values observed in these patients. Despite the fact that HM patients had higher TPO levels compared to ITP patients, this difference was much smaller than the one that we reported in IPF. In fact, the estimated diagnostic accuracy for the differential diagnosis between HM and ITP was higher for the IPF (both in % and in absolute count) compared to TPO. In regard to the fact that no correlation was found between TPO levels with either platelet counts or IPF in HM, this suggests that increased IPF is not associated with increased thrombopoietic activity in HM.

The mechanisms underlying the increase of IPF in HM remain to be elucidated. The previously reported increase in platelet volume of around 50 to 100%^5, 25^ is probably not sufficient to explain the 200–300% increase of IPF in these patients. In addition, formation of platelet aggregates was ruled out by the microscopic evaluation of blood smears, and by a review of the scatter plots of IPF measurements from all patients with HM. The strong correlation between IPF measurements performed one year apart suggests that the higher RNA content of these platelets could be a stable characteristic associated with the cellular mechanisms of thrombocytopenia in these patients. Cellular mechanisms responsible for thrombocytopenia in HM are complex and dependent on the molecular etiology of each disease entity. Increased counts of immature platelets were described in an animal model of HM due to *RASA3* mutations in which platelet turnover is increased^26^. However, most of our patients presented with more classical forms of thrombocytopenia such as BSS and MYH9-related disorders in which thrombocytopenia is attributed to defects in proplatelet formation^27–29^. Interestingly, in MYH9 megakaryocytes, the physiological suppression of proplatelet extension exerted by the interaction with type I collagen is lost leading to premature or abnormal platelet release from the bone marrow^29^. We therefore speculate that the extremely high IPF values observed in most of our patients with HM are an expression of the premature release of platelets to the circulation. Interestingly, patients in whom the diagnosis of MYH9-related platelet disorders or BSS were confirmed by molecular or flow cytometry assays presented significantly higher IPF values than patients with a diagnosis of non-specified HMT. However, due to the limited number of patients in this subgroup analysis, these data should be considered preliminary. The confirmation of this hypothesis in future studies would provide a biological rationale to the use of IPF as a diagnostic tool in HM.

In accordance with the concept that ITP is a condition associated with suboptimal increases in TPO levels^23^, our analysis demonstrated significantly lower TPO levels in ITP patients compared to control subjects with normal platelet counts. Nevertheless, suboptimal TPO increase did not preclude the demonstration of a marked negative correlation between TPO levels and platelet counts in ITP (rho = −0.50), which was even stronger when only patients using steroid were evaluated (data not shown). This suggests that in ITP, increase in TPO levels is blunted by yet unknown mechanisms which are apparently improved by steroid therapy.

Our study presents limitations that need to be acknowledged, with lack of flow cytometry data being possibly the most relevant. As mentioned earlier divergent results were obtained when the IPF was measured in HM patients by flow cytometry in the study by Fabris *et al*.^13^ or using automated hematological analyzers, as in the study by Myiazaki^14^ and ours. Few studies have formally evaluated the correlation of IPF measurements by these two methods but in two of the most recent ones, this correlation was limited to ITP patients^11, 30^. Considering the challenges of standardizing platelet flow cytometry analyses compared to a CBC, we believe that it is fair to state that the former method should not be regarded as a gold-standard for IPF measurement. In fact, the vast majority of studies addressing other uses of IPF do not perform parallel flow cytometry analysis. Moreover, the concordance of our results with those from Myiazaki *et al*. in two independent populations supports the conclusion that IPF is increased in HM, possibly showing the premature release of platelets into the circulation. In addition, the analyzer used in our study measures MPV by impedance, which is a method that in patients with HM is known to report MPV results in less than half of the cases due to abnormalities in the platelet distribution curve^4, 5^. This technical limitation was also present in our study, and precluded some interesting analyses such as the comparison of the estimated diagnostic accuracy of IPF and MPV for the differential diagnosis between HM and ITP, and the evaluation of the combined performance of IPF and MPV in this context. On the other hand, it illustrates the challenges of using the MPV as a diagnostic test for HM, highlighting the importance of additional assays such as the IPF. Interestingly, when MPV values from our patients obtained from the same sample in a different hematological analyzer (Advia 2120) were used, the estimated diagnostic accuracy for the differential diagnosis of HM and ITP (AUC = 0.64; 95%CI 0.50–0.79; P = 0.02) was lower than that of the IPF.

In conclusion, our results validate previous demonstrations that IPF is increased in HM in an independent and larger population of patients. We also demonstrate the reproducibility of this method, thus supporting the feasibility of its incorporation in the diagnostic approach of HM. Given the easy accessibility to this parameter for routine clinical laboratories, and the importance of differentiating HM and ITP, further studies are warranted to the incorporation of the IPF in diagnostic algorithms of HM, and to further investigate new questions raised by our study such as the mechanisms of IPF elevation in HM.
